# Cardiopulmonary Bypass Down-Regulates NOD Signaling and Inflammatory Response in Children with Congenital Heart Disease

**DOI:** 10.1371/journal.pone.0162179

**Published:** 2016-09-13

**Authors:** Qinghua Yang, Jianyi Liao, Jie Huang, Yi Ping Li, Shungen Huang, Huiting Zhou, Yi Xie, Jian Pan, Yanhong Li, Jiang Huai Wang, Jian Wang

**Affiliations:** 1 Department of Pediatric Surgery, Children’s Hospital of Soochow University, Suzhou, China; 2 Department of Pediatric Cardiology, Children’s Hospital of Soochow University, Suzhou, China; 3 Institute of Pediatric Research, Children’s Hospital of Soochow University, Suzhou, China; 4 Department of Academic Surgery, University College Cork, Cork University Hospital, Cork, Ireland; Universitat Hohenheim, GERMANY

## Abstract

In the present study, we aimed to examine the impact of cardiopulmonary bypass (CPB) on expression and function of NOD1 and NOD2 in children with congenital heart disease (CHD), in an attempt to clarify whether NOD1 and NOD2 signaling is involved in the modulation of host innate immunity against postoperative infection in pediatric CHD patients. Peripheral blood samples were collected from pediatric CHD patients at five different time points: before CPB, immediately after CPB, and 1, 3, and 7 days after CPB. Real-time PCR, Western blot, and ELISA were performed to measure the expression of NOD1 and NOD2, their downstream signaling pathways, and inflammatory cytokines at various time points. Proinflammatory cytokine IL-6 and TNF-α levels in response to stimulation with either the NOD1 agonist Tri-DAP or the NOD2 agonist MDP were significantly reduced after CPB compared with those before CPB, which is consistent with a suppressed inflammatory response postoperatively. The expression of phosphorylated RIP2 and activation of the downstream signaling pathways NF-κB p65 and MAPK p38 upon Tri-DAP or MDP stimulation in PBMCs were substantially inhibited after CPB. The mRNA level of NOD1 and protein levels of NOD1 and NOD2 were also markedly decreased after CPB. Our results demonstrated that NOD-mediated signaling pathways were substantially inhibited after CPB, which correlates with the suppressed inflammatory response and may account, at least in part, for the increased risk of postoperative infection in pediatric CHD patients.

## Introduction

Congenital heart disease (CHD) is the most common congenital malformation [1] with a high morbidity and mortality rate in infants and young children [2]. In recent years, the rapid development in medical technology such as cardiopulmonary bypass surgery (CPB) and postoperative myocardial protection and support have improved the survival rate of children with CHD. However, CPB predisposes to postoperative infections, which predominately affect the postoperative rehabilitation of children with CHD. The rate of post-CPB infection in pediatric CHD patients is approximately 16–31% [3, 4], and the exact mechanism underlying the increased risk of infection in this population remains unclear.

Early infection is recognized by innate immune cells based on the detection of pathogen-associated molecular patterns (PAMPs), which initiates the inflammatory response and host defense. Pattern recognition receptors (PRRs) play an important role in this process, whereas the dysfunction in PRRs predisposes host to a variety of microbial pathogens. The nucleotide-binding and oligomerization domain (NOD)-like receptors (NLRs) are the recently discovered class of PRRs, which are located in the intracellular cytoplasm and play a key role in recognizing microbial pathogens and mediating inflammation [5, 6]. Both NOD1 and NOD2 are the most representative of NLR family and identify different core motifs of bacterial peptidoglycan (PGN) derivatives [7–9]. Although NOD1 and NOD2 sense different ligands of bacterial origin, they both recruit and interact with the adaptor protein receptor-interacting protein kinase 2 (RIP2) [10] and subsequently activate the same downstream signaling pathways, the NF-κB pathway and mitogen-activated protein kinase (MAPK) pathway, which ultimately leads to a rapid production of inflammatory cytokines including TNF-α and IL-6, thereby initiating the innate immune and inflammatory responses to eliminate microbial pathogens and prevent bacterial infection [11–13].

Our previous work has shown that CPB substantially suppresses Toll-like receptor (TLR)-mediated signal transduction pathways, which leads to a compromised inflammatory response in children with CHD [3]. In the present study, we further examined the influence of CPB on activation of NOD1- and NOD2-mediated signaling pathways and production of inflammatory cytokines in peripheral blood samples and peripheral blood mononuclear cells (PBMCs) collected from pediatric CHD patients before and after CPB.

## Materials and Methods

### Study subjects

This study was approved by the Institutional Research Ethics Committee of Affiliated Children’s Hospital and Soochow University (Suzhou, China) for clinical investigation and the written informed consent was obtained from parents of the recruited children prior to enrollment. All experiments and procedures involving human subjects were conducted according to the principles in the Declaration of Helsinki. A total of 44 children who diagnosed with CHD to undergo elective CPB for surgical repair between May 2014 and December 2014 were randomly selected for inclusion in this study. The exclusion criteria included diagnosis of a genetic syndrome, active infection or inflammation preoperatively, documented immunodeficiency, and refusal of consent. Standardized anesthesia with intravenous injection of propofol, remifentanil, and cis-atracurium for induction of anesthesia, and sevoflurane, remifentanil, and cis-atracurium for maintaining anesthesia were used. Multifunctional life monitor (CILIN-508, Japan) was used for operative monitoring. Clinical information and patient’s data are summarized in Table 1.

**Table 1:** Clinical information and data.

Ages (year)	1.81 ± 1.93
male/female	20/24
weight (kg)	10.08 ± 3.49
Ventricular septal defect	28
Atrial septal defect	11
Tetralogy of Fallot	5
CPB time (min)	81.64 ± 27.53
Aortic clamping time (min)	44.48 ± 19.67
PICU time (min)	1.97 ± 0.26

Note: age, weight, CPB time, aortic clamping time, PICU time are all represented as mean ± standard deviation (x ± SD).

### Blood sampling

Blood samples (2.0 ml) were obtained from arterial or central venous lines at the following time points: pre-CPB (Tc), immediately post-CPB (T0), at day 1 (T1), day 3 (T3), and day 7 (T7) post-CPB. At each time point, freshly heparinized blood samples were collected and centrifuged. PBMCs were isolated by density gradient centrifugation using lymphocyte separation medium (Hao Yang Biological Products Technology, Tianjin, China) for subsequent RNA and protein extraction.

### Ex vivo stimulation and cytokine measurement

A 50 μl sample of the heparinized whole blood diluted with 450 μl of RPMI 1640 and isolated PBMCs were stimulated *ex vivo* with PBS as the control, Tri-DAP (100 ng/ml) (InvivoGen, San Diego, CA, USA) or MDP (100 ng/ml) (InvivoGen) for 4 and 12 hrs at 37°C in a humidified 5% CO_2_ atmosphere. The stimulated samples were immediately centrifuged at 3,000 rpm for 20 min at 4°C, and cell-free supernatants were collected and stored at -80°C until analysis. TNF-α and IL-6 concentrations in the supernatants were assessed by ELISA (BD Biosciences, San Jose, CA, USA).

### Quantitative real-time RT-PCR

Total RNA was extracted from PBMCs isolated from pediatric CHD patients at different time points (Tc, T0, T1, T3, and T7) using TRIzol reagent (Invitrogen Life Technologies, Paisley, Scotland, UK) and was reverse-transcribed into cDNA using the reverse transcriptase (Promega Corporation, Madison, WI, USA) according to the manufacturer’s instructions. Fluorescent quantitative real-time RT-PCR was performed for amplification of cDNA using a LightCyler480 (Roche Applied Science, Basel, Switzerland), and all data were collected and analyzed. The following primers were used: human NOD1 (sense-5’-TCCAAAGCCAAACAGAAACTC-3’ and antisense-5’-CAGCATCCAGATGAACGTG-3’); human NOD2 (sense-5’-GAAGTACATCCGCACCGAG-3’ and antisense-5’-GACACCATCCATGAGAAGACAG-3’); human β-actin (sense-5’-TGACAGGATCGAGAAGGAGA-3’ and antisense-5’-CGCTCAGGAGGAGCAATG-3’). The NOD1 and NOD2 mRNA expression was normalized with the housekeeping gene β-actin.

### Western blot analysis

PBMCs isolated from pediatric CHD patients at different time points (Tc, T0, T1, T3, and T7) were lysed in cell lysis buffer (Cell Signaling Technologies, Beverly, MA, USA) on ice for 30 min. For measurement of RIP2, NF-κB p65, and MAPK p38, PBMCs were stimulated with PBS as the control, Tri-DAP (100 ng/ml), or MDP (100 ng/ml) for 30 min and then lysed in cell lysis buffer (Cell Signaling Technology) supplemented with PMSF (1 mM) and protease inhibitor cocktail (Roche Diagnostics GmbH, Mannheim, Germany) on ice for 30 min. The resultant lysates were centrifuged and supernatants containing the cytoplasmic proteins were collected. Protein concentration was measured using a micro BCA protein assay (Pierce, Rockford, IL, USA). Equal amounts of protein extracts were separated on SDS-polyacrylamide gels and *trans*-blotted onto polyvinylidene difluoride membranes (Schleicher & Schuell, Dassel, Germany). The membranes were blocked for 1 h at room temperature with PBS containing 0.05% Tween-20 and 5% nonfat milk and probed overnight at 4°C with the appropriate primary mAbs anti-NOD1 (Abcam, Cambridge, MA, USA), anti-NOD2 (Abcam), anti-total RIP2 (Cell Signaling Technology), anti-phosphorylated RIP2 at Ser^176^ (Cell Signaling Technology), anti–phosphorylated anti-total p65 (Cell Signaling Technology), anti-phosphorylated p65 at Ser^536^ (Cell Signaling Technology), anti-total p38 (Cell Signaling Technology), and anti-phosphorylated p38 at Thr^180^/Tyr^182^ (Cell Signaling Technology). Blots were then incubated with the appropriate HRP-conjugated secondary Abs (Dako, Cambridge, UK) at room temperature for 1 hr, developed with SuperSignal chemiluminescence substrate (Pierce) and captured with LAS-3000 imaging system (Fujifilm, Tokyo, Japan).

### Statistical analysis

All data are presented as mean ± standard deviation (SD). Statistical analysis was performed using GraphPad software, version 5.01 (Prism, La Jolla, CA, USA). Comparison between groups was carried out using one-way ANOVA. Differences were judged statistically significant when the p value was less than 0.05.

## Results

### CPB attenuates NOD agonist-stimulated inflammatory cytokine production

**Fig 1:**
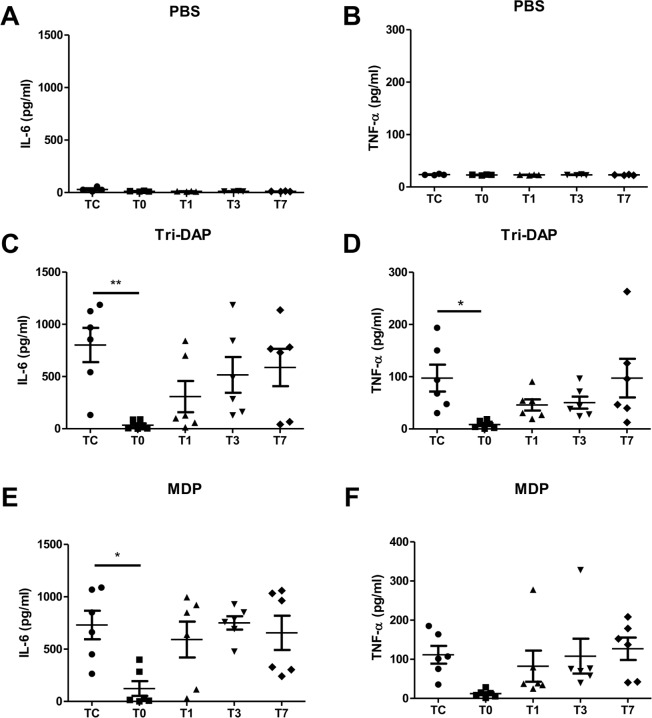
CPB attenuates the NOD1 and NOD2 agonist-stimulated IL-6 and TNF-α production in whole blood. Heparinized whole blood samples were collected from pediatric CHD patients before CPB (Tc), immediately after CPB (T0), at day 1 (T1), day 3 (T3), and day 7 (T7) after CPB, and stimulated with PBS as the control (A and B), the NOD1 agonist Tri-DAP (100 ng/ml) (C and D), and the NOD2 agonist MDP (100 ng/ml) (E and F) for 4 hrs. IL-6 and TNF-α concentrations in the supernatants were assessed by ELISA. *p<0.05, **p<0.01 compared with Tc.

**Fig 2:**
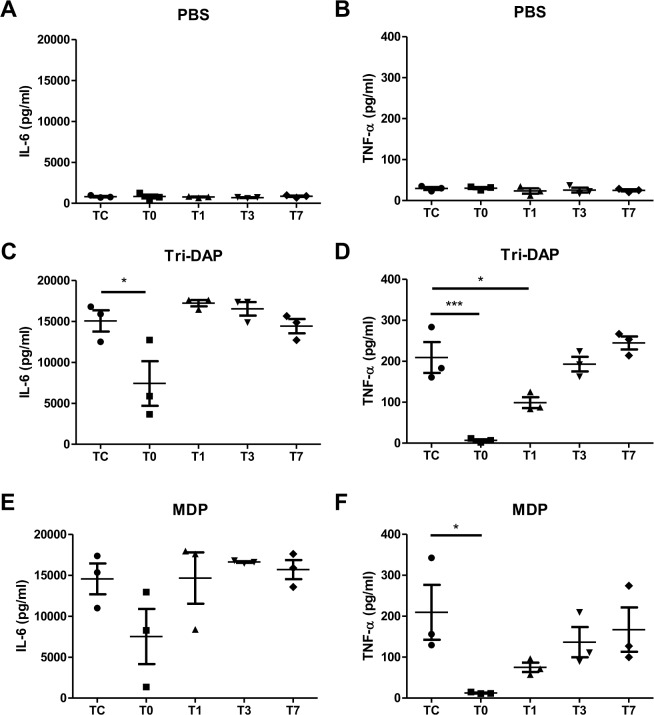
CPB attenuates the NOD1 and NOD2 agonist-stimulated IL-6 and TNF-α production in PBMCs. Heparinized whole blood samples were collected from pediatric CHD patients before CPB (Tc), immediately after CPB (T0), at day 1 (T1), day 3 (T3), and day 7 (T7) after CPB for isolation of PBMCs. PBMCs were stimulated with PBS as the control (A and B), the NOD1 agonist Tri-DAP (100 ng/ml) (C and D), and the NOD2 agonist MDP (100 ng/ml) (E and F) for 12 hrs. IL-6 and TNF-α concentrations in the supernatants were assessed by ELISA. *p<0.05, ***p<0.001 compared with Tc.

Peripheral blood samples and PBMCs collected from pediatric CHD patients before and after CPB were subjected to *ex vivo* stimulation with PBS as the control, the NOD1 agonist Tri-DAP, and the NOD2 agonist MDP to assess the inflammatory cytokine response. There was neglectable or very little production of proinflammatory cytokines IL-6 and TNF-α in blood samples (Fig 1A and 1B) and PBMCs (Fig 2A and 2B) at all time points after PBS treatment; however, stimulation with either Tri-DAP or MDP resulted in a substantially enhanced production of IL-6 and TNF-α before CPB in both blood samples (Fig 1C–1F) and PBMCs (Fig 2C–2F). A strong attenuation in Tri-DAP-stimulated release of IL-6 (*p*<0.05, *p*<0.01) and TNF-α (*p*<0.05, *p*<0.001) was observed in blood samples (Fig 1C and 1D) and PBMCs (Fig 2C and 2D) immediately post-CPB compared with those pre-CPB. Tri-DAP-stimulated IL-6 and TNF-α levels began to increase at day 1 post-CPB and returned to the preoperative levels at day 7 post-CPB (Fig 1C and 1D) (Fig 2C and 2D). MDP-stimulated IL-6 release (Fig 1E), but not TNF-α (Fig 1F), was markedly suppressed in blood samples immediately post-CPB (*p*<0.05 versus the pre-CPB IL-6 level) and returned to the preoperative level at day 1 post-CPB, whereas MDP-stimulated TNF-α (Fig 2F) release, but not IL-6 (Fig 2E), was markedly suppressed in PBMCs immediately post-CPB (*p*<0.05 versus the pre-CPB TNF-α level) and began to increase at day 1 post-CPB.

### CPB down-regulates NOD1 and NOD2 mRNA and protein expression

**Fig 3:**
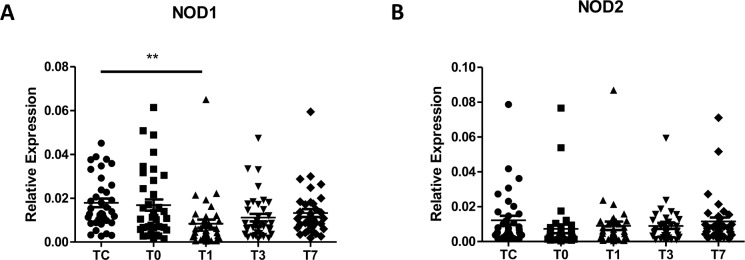
CPB down-regulates NOD1, but not NOD2, mRNA expression. Heparinized whole blood samples were collected from pediatric CHD patients before CPB (Tc), immediately after CPB (T0), at day 1 (T1), day 3 (T3), and day 7 (T7) after CPB for isolation of PBMCs. Total RNA was extracted and subjected to quantitative real-time RT-PCR for analysis of NOD1 (A) and NOD2 (B) mRNA expression. **p<0.01 compared with Tc.

**Fig 4:**
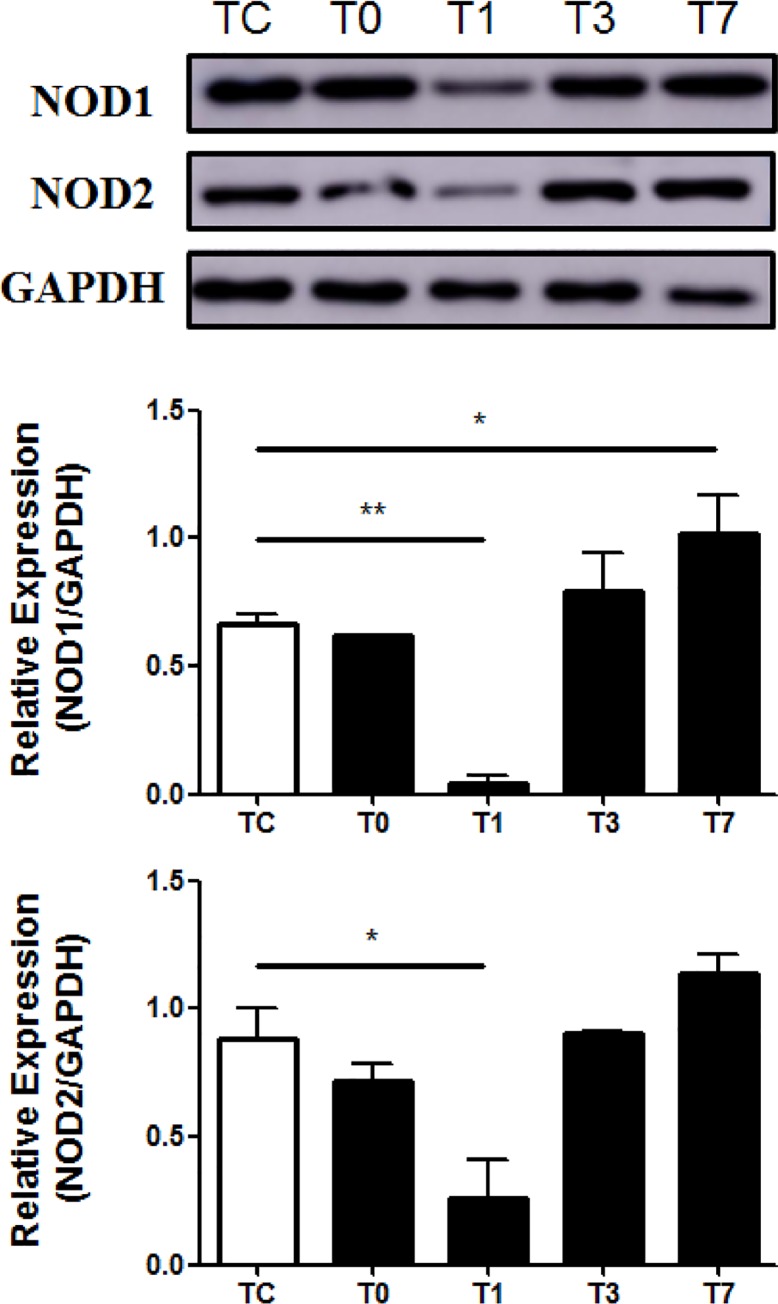
CPB suppresses both NOD1 and NOD2 protein expression. Heparinized whole blood samples were collected from pediatric CHD patients before CPB (Tc), immediately after CPB (T0), at day 1 (T1), day 3 (T3), and day 7 (T7) after CPB for isolation of PBMCs. Cytoplasmic proteins were extracted and subjected to Western blot analysis for NOD1 and NOD2 protein expression. Data shown represent one experiment from a total of three separate experiments. Density ratios of NOD1/GAPDH and NOD2/GAPDH were quantified by densitometry analysis (n = 3). *p<0.05, **p<0.01 compared with Tc.

Next, we examined the influence of CPB on NOD1 and NOD2 mRNA and protein expression in PBMCs collected from pediatric CHD patients before and after CPB. NOD1 mRNA expression in pediatric CHD patients was significantly down-regulated at day 1 post-CPB compared with the preoperative level (*p*<0.01) and returned to preoperative levels from day 3 post CPB (Fig 3A), whereas NOD2 mRNA expression was reduced immediately post-CPB, but was not significantly different compared with the preoperative level (Fig 3B). Moreover, a strong down-regulation of NOD1 protein expression was found in PBMCs of pediatric CHD patients at day 1 post-CPB (Fig 4), whereas substantially reduced expression of NOD2 protein was also observed immediately post-CPB and at day 1 post-CPB in PBMCs of pediatric CHD patients (Fig 4).

### CPB suppresses expression of phosphorylated RIP2 and activation of downstream MAPK p38 and NF-κB p65

**Fig 5:**
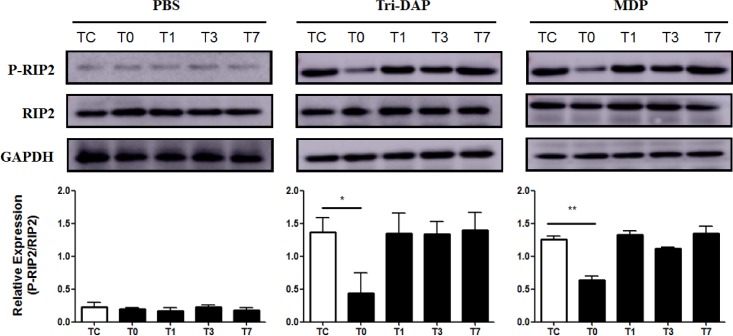
CPB down-regulates expression of the phosphorylated RIP2. Heparinized whole blood samples were collected from pediatric CHD patients before CPB (Tc), immediately after CPB (T0), at day 1 (T1), day 3 (T3), and day 7 (T7) after CPB for isolation of PBMCs. PBMCs were stimulated with PBS as the control, the NOD1 agonist Tri-DAP (100 ng/ml), and the NOD2 agonist MDP (100 ng/ml) for 30 min. Cytoplasmic proteins were extracted and subjected to Western blot analysis for RIP2 protein expression. Data shown represent one experiment from a total of three separate experiments. P-RIP2 = phosphorylated RIP2. Density ratio of P-RIP2/RIP2 was quantified by densitometry analysis (n = 3). *p<0.05, **p<0.01 compared with Tc.

**Fig 6:**
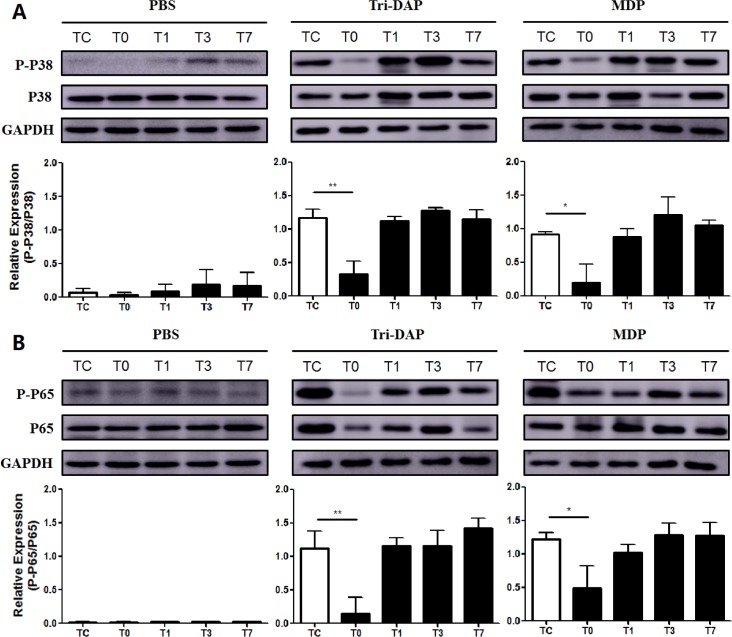
CPB suppresses activation of both NF-κB p65 and MAPK p38. Heparinized whole blood samples were collected from pediatric CHD patients before CPB (Tc), immediately after CPB (T0), at day 1 (T1), day 3 (T3), and day 7 (T7) after CPB for isolation of PBMCs. PBMCs were stimulated with PBS as the control, the NOD1 agonist Tri-DAP (100 ng/ml), and the NOD2 agonist MDP (100 ng/ml) for 30 min. Cytoplasmic proteins were extracted and subjected to Western blot analysis for MAPK p38 (A) and NF-κB p65 (B) phosphorylation. Data shown represent one experiment from a total of three separate experiments. P-P38 = phosphorylated MAPK p38, P-P65 = phosphorylated NF-κB p65. Density ratios of P-P38/P38 (A) and P-P65/P65 (B) were quantified by densitometry analysis (n = 3). *p<0.05, **p<0.01 compared with Tc.

We further determined whether CPB affects expression of RIP2, the adaptor protein of NOD signaling, and phosphorylation of NF-κB p65 and MAPK p38, the downstream pathways of NOD signaling. Up-regulation of phosphorylated RIP2 in response to stimulation with either the NOD1 agonist Tri-DAP- or the NOD2 agonist MDP in PBMCs was strongly inhibited immediately post-CPB in comparison with phosphorylated RIP2 levels before CPB (Fig 5). Furthermore, stimulation with Tri-DAP and MDP led to a strong activation of MAPK p38 (Fig 6A) and NF-κB p65 (Fig 6B) in PBMCs before CPB; however, substantial suppression in either Tri-DAP- or MDP-stimulated phosphorylation of MAPK p38 (Fig 6A) and NF-κB p65 (Fig 6B) was observed in PBMCs collected immediately post-CPB compared with those collected before CPB. Both Tri-DAP- and MDP-stimulated MAPK p38 and NF-κB p65 phosphorylation was gradually returned to the preoperative level from day 1 post-CPB (Fig 6A and 6B).

## Discussion

Dysregulation of NOD signaling has been linked to predisposition to a variety of microbial infection, which is associated closely with an impaired inflammatory response [6, 9, 11–12]. In the present study, we found that the NOD1 agonist Tri-DAP-stimulated IL-6 and TNF-α release in both blood samples and PBMCs collected from pediatric CHD patients was markedly reduced immediately after CPB, whereas the NOD2 agonist MDP-stimulated IL-6 release in blood samples and MDP-stimulated TNF-α release in PBMCs were also substantially decreased immediately after CPB, indicating that CPB suppresses NOD signaling-mediated inflammatory response in pediatric CHD patients.

NOD signaling-mediated production and release of inflammatory cytokines is primarily related to the expression of NOD including NOD1 and NOD2, their adaptor protein RIP2, and activation of NOD-mediated downstream signaling pathways. We first assessed NOD1 and NOD2 mRNA and protein expression in PBMCs of pediatric CHD patients before and after CPB. We observed that levels of NOD1 mRNA and protein expression were substantially reduced at day 1 after CPB, whereas NOD2 protein levels were also markedly reduced immediately after CPB and at day 1 after CPB. These results indicate that suppressed expression of NOD1 and NOD2 after CPB correlates with diminished IL-6 and TNF-α production upon NOD activation, suggesting that down-regulation of NOD1 and NOD2 is responsible, at least in part, for a compromised inflammatory response observed in pediatric CHD patients after CPB.

The adaptor protein RIP2 and the downstream signal pathways NF-κB p65 and MAPK p38 phosphorylation play key roles in the initiation and activation of NOD signaling-mediated inflammatory responses [6, 12–13]. We next examined the expression of RIP2 and phosphorylation of NF-κB p65 and MAPK p38 at different time points before and after CPB. We found substantially down-regulated expression of phosphorylated RIP2 and markedly suppressed activation of both NF-κB p65 and MAPK p38 in Tri-DAP-stimulated PBMCs immediately after CPB. MDP-stimulated expression of phosphorylated RIP2 and activation of NF-κB p65 and MAPK p38 were also strongly inhibited in PBMCs immediately after CPB. These results indicate that down-regulated activation of RIP2, NF-κB p65, and MAPK p38 in response to stimulation with either the NOD1 agonist Tri-DAP or the NOD2 agonist MDP may be the underlying mechanism(s) responsible for the dysregulated inflammatory response, as represented by attenuated TNF-α and IL-6 production after CPB in pediatric CHD patients. Recent studies further suggest that NOD signal can be transduced independent of RIP2 pathway, whereas MDP can directly interact with NLR family pyrin domain containing 1 (NLRP1) and NLRP3 without activating RIP2 [14–16]. Thus, CPB might affect the activation of multiple NOD downstream molecules; however, the exact mechanisms and synergistic effects between these downstream molecules remain to be elucidated.

Our previous work has shown that CPB during pediatric cardiac surgery leads to a substantially suppressed inflammatory response to either gram-negative or gram-positive bacterial component stimulation, which is associated with CPB-induced attenuation in TLR-mediated signal transduction pathways [3]. In the present study, we further demonstrated that CPB strongly inhibited NOD-mediated intracellular signaling pathways, thus attenuating proinflammatory cytokine production in response to stimulation with both NOD1 and NOD2 agonists. These findings suggest that CPB-induced compromised inflammatory response is a common phenomenon in pediatric CHD patients during cardiac surgery, which may contribute, at least in part, to an increased risk of development of postoperative infection in these patients.

## Abbreviations

CPB: cardiopulmonary bypass
CHD: congenital heart disease
MAPK: mitogen-activated protein kinase
NLRs: nucleotide-binding and oligomerization domain (NOD)-like receptors
NLRP1: NLR family pyrin domain containing 1
PAMPs: pathogen-associated molecular patterns
PGN: peptidoglycan
PBMCs: peripheral blood mononuclear cells
PRRS: pattern recognition receptors
RIP2: receptor-interacting protein kinase 2
TLRs: Toll-like receptors
